# National trends in hospitalization and mortality rates for patients with HIV, HCV, or HIV/HCV coinfection from 1996–2010 in the United States: a cross-sectional study

**DOI:** 10.1186/1471-2334-14-536

**Published:** 2014-10-10

**Authors:** Christine U Oramasionwu, Joshua C Toliver, Terence L Johnson, Heather N Moore, Christopher R Frei

**Affiliations:** Division of Pharmaceutical Outcomes and Policy, University of North Carolina, UNC Eshelman School of Pharmacy, 2215 Kerr Hall, Chapel Hill, NC 27599-7573 USA; The University of Texas at Austin, College of Pharmacy, Austin, TX USA; The University of Texas Health Science Center San Antonio, School of Medicine, San Antonio, TX USA

**Keywords:** HIV, HCV, Coinfection, Hospitalization, Health care utilization

## Abstract

**Background:**

The comparative impact of chronic viral monoinfection versus coinfection on inpatient outcomes and health care utilization is relatively unknown. This study examined trends, inpatient utilization, and hospital outcomes for patients with HIV, HCV, or HIV/HCV coinfection.

**Methods:**

Data were from the 1996–2010 National Hospital Discharge Surveys. Hospitalizations with primary ICD-9-CM codes for HIV or HCV were included for HIV and HCV monoinfection, respectfully. Coinfection included both HIV and HCV codes. Demographic characteristics, select comorbidities, procedural interventions, average hospital length of stay (LOS), and discharge status were compared by infection status (HIV, HCV, HIV/HCV). Annual disease estimates and survey weights were used to generate hospitalization rates.

**Results:**

~6.6 million hospitalizations occurred in patients with HIV (39%), HCV (56%), or HIV/HCV (5%). The hospitalization rate (hospitalizations per 100 persons with infection) decreased in the HIV group (29.8 in 1996; 5.3 in 2010), decreased in the HIV/HCV group (2.0 in 1996; 1.5 in 2010), yet increased in the HCV group (0.2 in 1996; 0.9 in 2010). Median LOS from 1996 to 2010 (days, interquartile range) decreased in all groups: HIV, 6 (3–10) to 4 (3–8); HCV, 5 (3–9) to 4 (2–6); HIV/HCV, 6 (4–11) to 4 (2–7). Age-adjusted mortality rates decreased for all three groups. The rate of decline was least pronounced for those with HCV monoinfection.

**Conclusion:**

Hospitalizations have declined more rapidly for patients with HIV infection (including HIV/HCV coinfection) than for patients with HCV infection. This growing disparity between HIV and HCV underscores the need to allocate more resources to HCV care in hopes that similar large-scale improvements can also be accomplished for patients with HCV.

**Electronic supplementary material:**

The online version of this article (doi:10.1186/1471-2334-14-536) contains supplementary material, which is available to authorized users.

## Background

Human Immunodeficiency Virus (HIV) monoinfection and hepatitis C virus (HCV) monoinfection have been the subject of much research over the past two decades; however, HIV/HCV coinfection is a growing medical concern in the U.S. [1]. Combination HIV antiretroviral therapy and combination HCV antiviral therapy have been recommended since the 1990s, as the respective treatment regimens greatly reduce patient morbidity and mortality [2, 3]. While HIV antiretroviral and HCV antiviral therapies are widely recommended for use in patients with HIV/HCV coinfection [4], these patients continue to experience poorer health outcomes than their counterparts with monoinfection. For instance, in the inpatient setting, patients with coinfection are at increased risk for accelerated progression of liver disease and increased rates of morbidity and mortality, as compared to patients with HIV or HCV monoinfection [5, 6].

However, since these combination therapies became available, few studies have documented how health care utilization patterns differ for patients with coinfection versus monoinfection. Thus, the burden that patients with coinfection place on the U.S. inpatient health care delivery system, as compared to patients with monoinfection, is relatively unknown. This study chronicled and compared inpatient health care utilization, including hospitalization rates, median length of hospital stay (LOS), and patient mortality rates, for patients with HIV, HCV, or HIV/HCV coinfection.

## Methods

This was a nationally representative, retrospective, observational study using data from the National Hospital Discharge Survey (NHDS) for the years 1996 through 2010. These surveys contain data for approximately 270,000 hospital discharges per year from 500 general and pediatric hospitals while excluding federal, military, veteran affairs, and institutional hospitals (e.g., prison hospitals). Demographic data include patient age, sex, race, hospital geographic region, hospital bed size, and patient insurance status. The survey is available to the public and is a national representation of annual discharge records. Each record consists of up to seven International Classification of Diseases, Ninth Revision, Clinical Modification (ICD-9-CM) diagnosis codes, as well as up to four procedure codes.

Hospitalizations primarily related to HIV, HCV, or HIV/HCV were included in this analysis. The following ICD-9-CM codes were used: HIV infection (042, V08, 079.53) and HCV infection (070.41, 070.44, 070.51, 070.54, 070.70, 070.71). HIV monoinfection hospitalizations excluded HCV codes and HCV monoinfection hospitalizations excluded HIV codes. HIV/HCV coinfection hospitalizations included those that had ICD-9-CM codes for both HIV and HCV. Procedural interventions were identified using ICD-9-CM procedure codes. Patients <15 years of age at the time of hospitalization were excluded from this analysis. Variables from the NHDS included patient demographics (patient age at time of hospitalization [years], race, gender, insurance status) as well as hospitalization characteristics (discharge diagnosis, hospital geographic region in the U.S., year of hospitalization, hospital discharge status, and LOS [days]). Other comorbidities, such as hepatitis B virus infection, illicit drug use, alcohol use, and Charlson co-morbidity index scores, were determined based on other diagnosis codes.

All data were analyzed using JMP 9.0® (SAS Corp, Cary, NC). Normally distributed continuous variables were expressed as means (±standard deviation) and categorical variables as percentages. Demographic characteristics and select comorbidities were compared by patient infection status (HIV, HCV, and HIV/HCV). The chi-square test was used for comparisons of categorical variables and the Student’s t-test was used for comparison of continuous variables. Annual population-level estimates for HIV monoinfection, HCV monoinfection, and HIV/HCV coinfection were computed to generate annual hospitalization rates. The U.S. Standard Million Population 2000 age distribution was used to calculate age-adjusted mortality rates. The University of North Carolina Office of Human Research Ethics determined that this project was not considered Human Subjects Research according to regulatory criteria; therefore, institutional review board approval was not needed.

## Results

**Figure 1:**
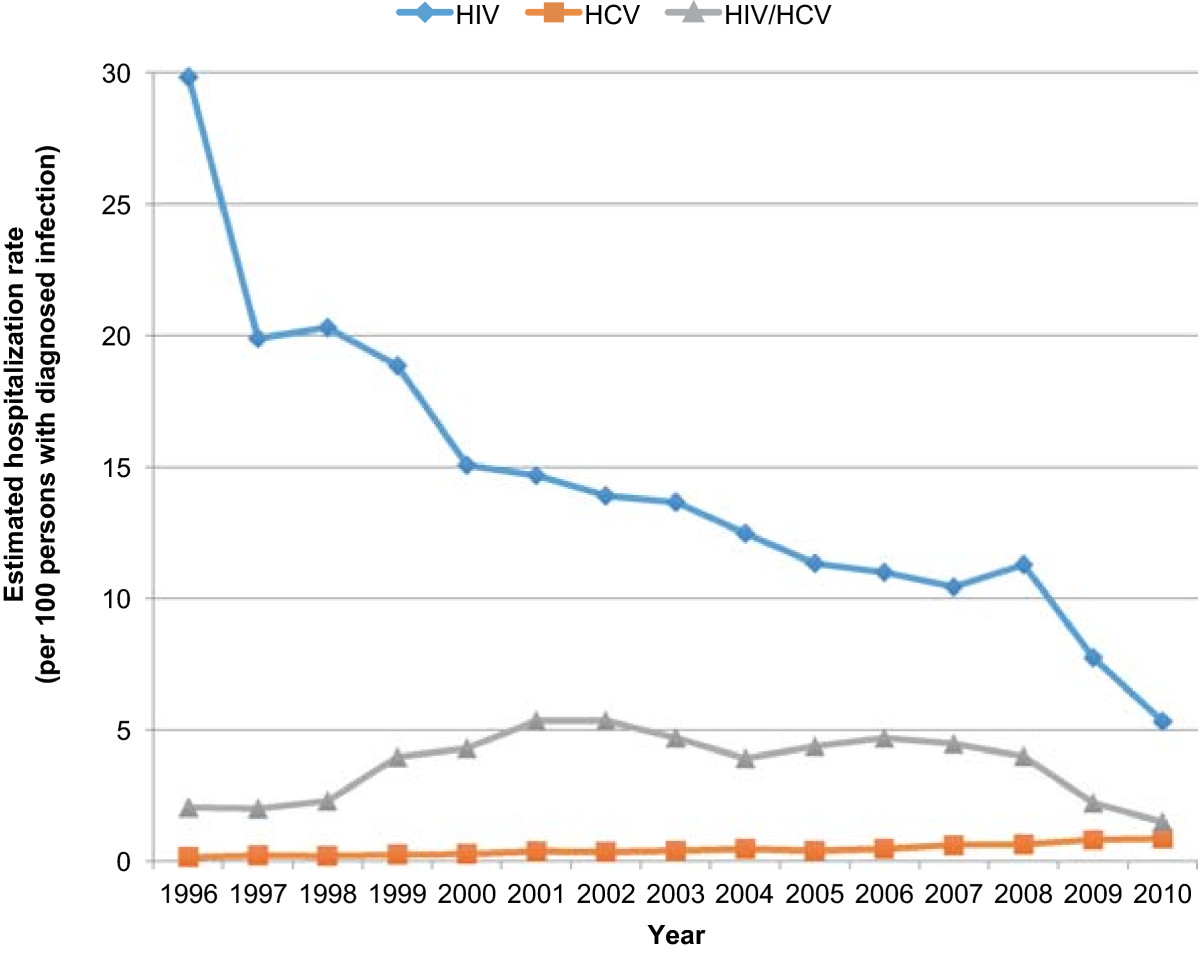
**Trends in hospitalization rates for HIV monoinfection, HCV monoinfection, or HIV/HCV coinfection.**

**Figure 2:**
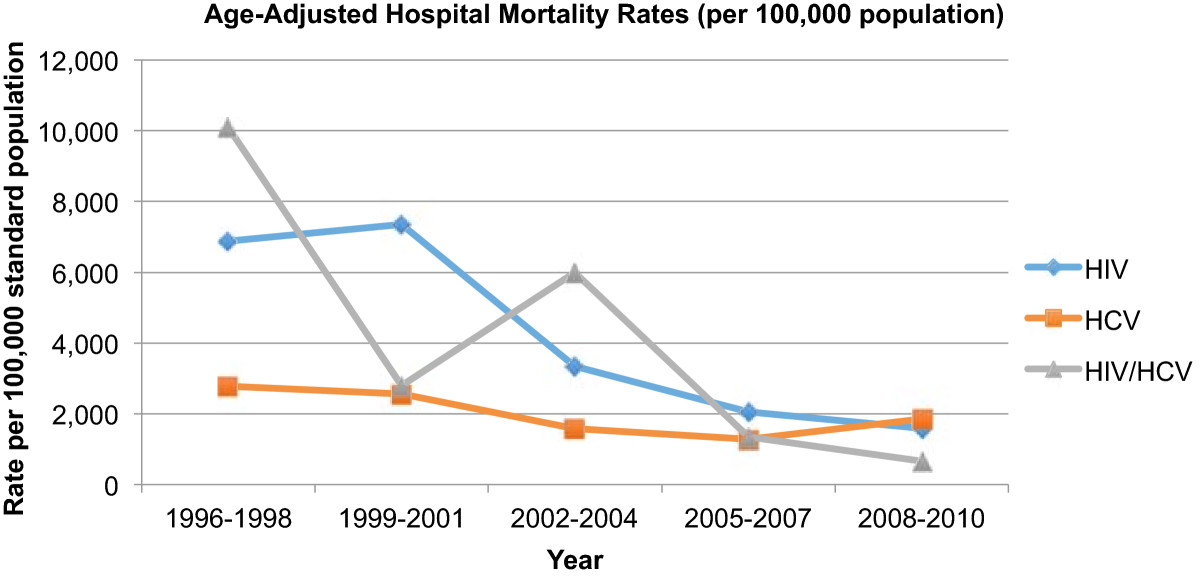
**Trends in age-adjusted hospital mortality rates for patients with HIV, HCV, or HIV/HCV.**

**Figure 3:**
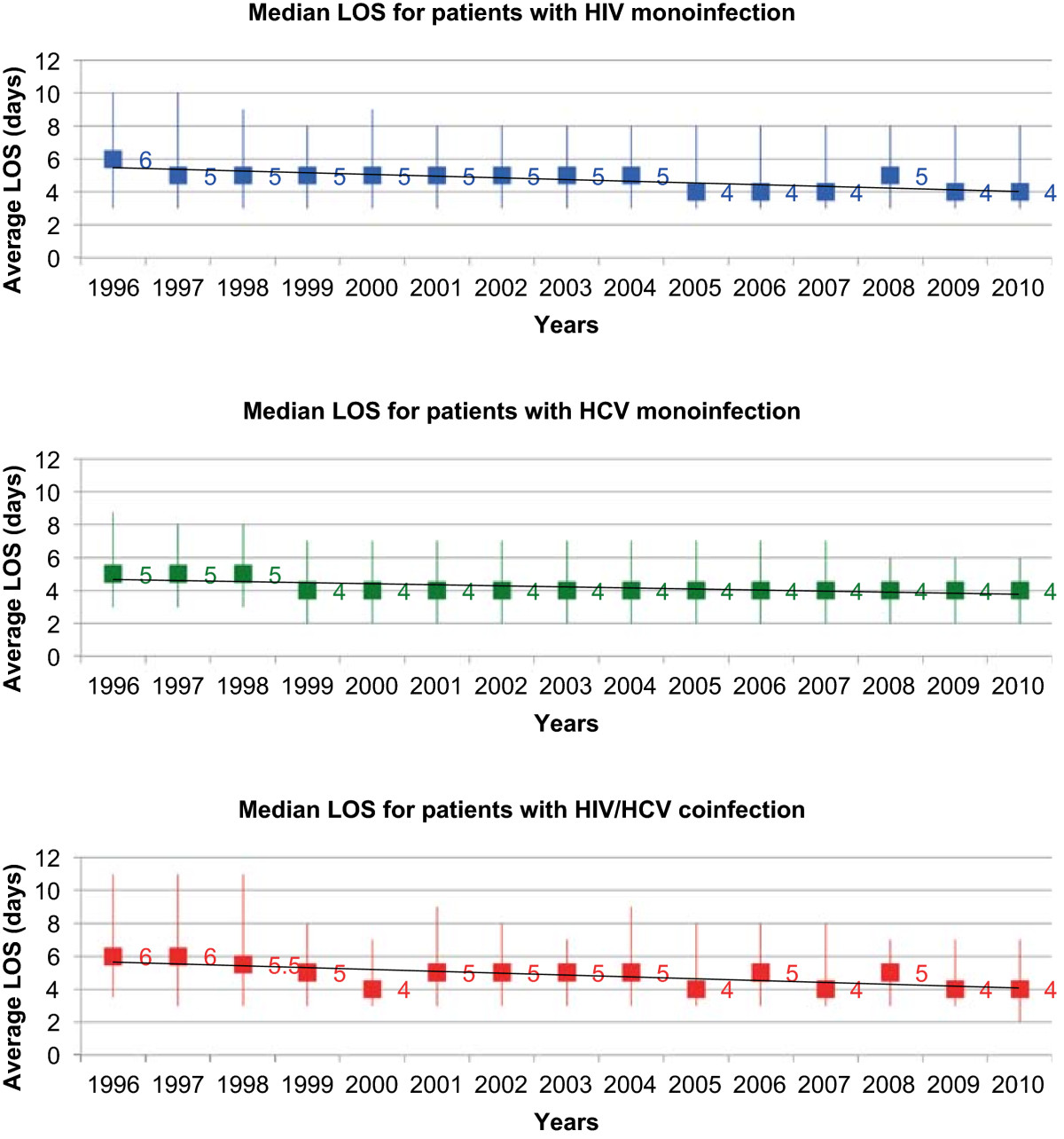
**Trends in average hospital length of stay from 1996–2010 for hospitalized patients with HIV, HCV, or HIV/HCV.**

Between 1996 and 2010, approximately 6.6 million hospitalizations met study criteria. Of these, 2,548,404 (39%) occurred in the group with HIV infection, 3,707,776 (56%) occurred in the group with HCV infection, and 317,307 (5%) occurred in the group with HIV/HCV coinfection (Table 1). Those with HCV were older in age, predominantly of white race, and had a higher percentage of females than those with HIV or HIV/HCV. In contrast, those with HIV were mostly of black race and were predominately from the southern United States. Those with coinfection were mostly of black race and were primarily Medicare insurance recipients. Comorbidities were distributed across the three groups (HIV, HCV, HIV/HCV) as follows: hepatitis B (2%, 5%, 10%), illicit drug use (15%, 17%, 24%), and alcohol use (9%, 19%, 13%). Overwhelmingly, inpatient care occurred in nonprofit hospitals (70%, 71% and 68% for HIV, HCV and HIV/HCV, respectfully). Most hospitalizations occurred in larger facilities. Inpatient procedures were distributed across the three groups (HIV, HCV, HIV/HCV) as follows: transfusion (8%, 9%, 8%), central line placement (8%, 8%, 8%), lumbar puncture (7%, 1%, 5%), bronchoscopy (5%, 1%, 3%), and upper GI endoscopy (4%, 6%, 4%). The majority of hospitalized patients were routinely discharged home (Table 2).Over the 15 years examined, the hospitalization rate (hospitalizations per 100 persons with infection) decreased in the HIV group (29.8 in 1996; 5.3 in 2010) and decreased slightly in the HIV/HCV group (2.0 in 1996; 1.5 in 2010) (Figure 1). The age-adjusted hospital mortality rates (per 100,000 population) over the same time interval decreased for all three groups; however, the rate of decline was least pronounced in the HCV group (Figure 2). The magnitude of the decreases ordered from greatest to least were as follows: HIV/HCV (10,098 in 1996; 661 in 2010), HIV (6,874 in 1996; 1,855 in 2010) and the smallest decrease for HCV (2,773 in 1996; 1,589 in 2010). From 1996 to 2010, median LOS (interquartile range) decreased in all groups: HIV group, 6 days (3–10) to 4 days (3–8); HCV group, 5 days (3–9) to 4 days (2–6); and HIV/HCV group, 6 days (4–11) to 4 days (2–7) (Figure 3).

**Table 1:** **Comparison of demographics, select comorbidities, and hospital characteristics for patients with HIV, HCV, or HIV/HCV**

Characteristic	HIV	HCV	HIV/HCV
	(n = 2,548,404)	(n = 3,707,776)	(n = 317,307)
***Demographics***	-	-	-
Age (yrs), mean ± SD	42 ± 10	50 ± 12	45 ± 8
Gender	-	-	-
Female	34%	39%	31%
Male	66%	61%	69%
Race/ethnicity	-	-	-
White	28%	57%	35%
Black	52%	19%	45%
Other	20%	24%	20%
Geographic region	-	-	-
South	44%	38%	38%
West	12%	24%	13%
Northeast	33%	24%	40%
Midwest	11%	14%	9%
Insurance	-	-	-
Private	19%	24%	12%
Medicare	24%	27%	22%
Medicaid	40%	30%	47%
Other/unknown	17%	19%	19%
***Comorbidities***	-	-	-
Hepatitis B virus	2%	5%	10%
Illicit drug use	15%	17%	24%
Alcohol use	9%	19%	13%
Charlson Score, mean ± SD	3.4 ± 2.6	1.5 ± 1.3	3.1 ± 2.6
***Hospital ownership***	-	-	-
Proprietary	7%	10%	7%
Government	23%	19%	25%
Nonprofit, including church	70%	71%	68%
***Hospital bedsize***	-	-	
<100 beds	7%	15%	5%
100-199	13%	21%	11%
200-299	16%	18%	16%
300-499	35%	27%	35%
≥500	29%	19%	32%

**Table 2:** **Discharge status for hospitalized patients with HIV, HCV, or HIV/HCV**

Discharge status	HIV	HCV	HIV/HCV
Routine/discharged home	73%	77%	72%
Left against medical advice	5%	4%	6%
Short-term care transfer	3%	4%	3%
Long-term care transfer	7%	6%	6%
Alive, not stated	5%	5%	7%
Dead	4%	3%	3%
Status not stated	3%	1%	3%

## Discussion

To our knowledge, this is the first study to chronicle and compare inpatient health care utilization for patients with HIV, HCV, or HIV/HCV over a long period of time (1996–2010). Our study reveals that there is a growing disparity in inpatient health care utilization for patients with HCV monoinfection, as compared to patients with HIV monoinfection or HIV/HCV coinfection. While age-adjusted patient mortality rates decreased for all three groups, the rate of decline in mortality was least pronounced in patients with HCV monoinfection. These findings suggest that patients with HCV infection disproportionately rely on inpatient care as compared to patients with HIV infection.

In general, those with HCV monoinfection were older in age and a greater proportion were female and of white race, while those with HIV (either monoinfection or coinfection) were younger in age and were predominately of black race. These demographic differences are consistent with results from studies by Ananthakrishnan *et al.*[5], which compared hospitalizations for patients with HIV, HCV, or HIV/HCV in the 2006 Nationwide Inpatient Sample (NIS), and by Backus *et al.*[7], which compared demographic characteristics in veterans with HIV monoinfection and veterans with HIV/HCV coinfection. As expected, our study found that the majority of hospitalizations occurred within nonprofit hospitals (bed size greater than 300) and were financed by public insurance. Hospitalizations with HIV or HCV monoinfection occurred more frequently within the southern region, whereas hospitalizations with coinfection occurred more frequently within the Northeast. Our study findings are representative of national trends in HIV and HCV populations.

One of the study objectives was to chronicle hospital outcomes, patient mortality, and hospital LOS, by infection status. Crude age-adjusted patient mortality decreased in all three groups, but the decrease in mortality was least pronounced for those with HCV monoinfection. Emerging data demonstrate rising mortality rates in persons with HCV [8, 9]. Using 1999–2007 death certificate data from the National Center for Health Statistics, Ly *et al.* examined and compared mortality rates attributable to HIV and to HCV [9]. Their analysis revealed that mortality rates during this time frame decreased for individuals with HIV, yet increased for individuals with HCV. There was a decrease of 0.21 deaths per 100,000 persons per year for HIV (*p =* 0.001), yet an increase in the annual age-adjusted mortality rate of 0.18 deaths per 100,000 persons per year for HCV (*p* = 0.002). By the end of their study period, HCV superseded HIV as a cause of death. While the decrease in average hospital LOS in the present study was less pronounced for those with HCV monoinfection, the decrease in hospital LOS throughout the study was relatively similar across all three groups. Gebo *et al.* studied hospitalization rates and intensive care unit utilization in a prospective cohort of individuals with HIV infection through the Johns Hopkins University AIDS Service in Baltimore, MD from 1995–2000 [6]. Mean hospital LOS was 7 days for both the HIV and HIV/HCV cohorts [6]. This is in contrast to Ananthakrishnan *et al.*’s 2006 NIS study, where average hospital LOS was slightly lower in patients with HIV/HCV coinfection than in patients with HIV monoinfection (-0.4 days, 95% CI -0.58 to -0.14) [5]. Despite past discordances in hospital LOS, this particular outcome appears to have improved for patients with monoinfection and for patients with coinfection in recent years.

This study adds to the evidence that HCV infection is placing a growing burden on the inpatient health care system. In the aforementioned study by Gebo *et al.*, hospitalization rates for the HIV monoinfected cohort were approximately 60 hospitalizations per 100 person-years in 1995 [6]. Yet by 1996, hospitalization rates had already decreased by one-third for these patients (*p* < 0.001), with rates remaining stable for the duration of the study. In contrast, rates increased significantly for the individuals with HIV/HCV coinfection, rising from 55.4 hospitalizations per 100 person-years in 1995 to 62.9 per 100 person-years in 2000 (*p* = 0.001) [6]. More recently, Sie *et al.* conducted a study of Los Angeles County residents who were hospitalized between 2007 and 2009 [10]. There was an increasing number of HCV-related hospitalizations in this region, leading the authors to conclude that HCV infection was becoming a growing economic burden on government resources due to rising costs of inpatient care required for these patients. Collectively, these findings emphasize the growing need for inpatient resource utilization in this patient population.

In 2007, Ly *et al.* documented a shift in mortality burden from HIV patients to HCV patients [9]. Prior investigations have documented differences in hospitalization rates for these patient populations. Ananthakrishnan *et al.*’s study of the 2006 NIS was a nationally representative evaluation of hospital outcomes for patients with HIV, HCV, or HIV/HCV [5]. Similar to the present study, the investigators compared hospitalizations rates across the three groups. Hospitalization rates in 2006 (hospitalizations per 100 persons) were as follows: HIV (19.9), HCV (14.8) and HIV/HCV (23.5). In comparison, hospitalization rates in 2006 (hospitalizations per 100 persons) for the present study were as follows: HIV (11), HCV (0.5), and HIV/HCV (4.7). Our lower rates are likely explained by the fact that we restricted our analysis to hospitalizations primarily related to HIV, HCV or HIV/HCV, whereas the previous study included all hospitalizations for individuals with these infections. A consistent result in both studies is that hospitalization rates for both HIV and HIV/HCV were greater than those for HCV. Unlike the Ananthakrishnan *et al.* study, we trended these rates over time to assess the differential burden of inpatient health care utilization for the different types of infection.

Our study also compared inpatient procedures as a component of inpatient health care utilization. Similar to Ananthakrishnan *et al.*’s NIS study, transfusion and central line placement were the most common inpatient interventions in all three groups [5]. Other procedural interventions varied consistently by infection status and are likely attributable to the need for diagnostic and prognostic testing for opportunistic infections. For example, the higher prevalence of lumbar puncture for those with HIV infection is likely explained by the need for cerebrospinal fluid evaluation for neurosyphilis or cryptococcal meningitis in these patients [11–13]. Similarly, the higher prevalence of bronchoscopy is likely explained by *Pneumocystis* pneumonia (PCP) work-up [13].

The uneven therapeutic advancements in HIV antiretroviral and HCV antiviral therapy may have led to disproportionate improvements in HIV patients compared to HCV patients. There have been numerous advances to HIV treatment, including the advent of highly active antiretroviral therapy (HAART) nearly two decades ago [2]. In contrast, combination therapy with interferon and ribavirin has been the standard therapy for HCV treatment for many years, despite the limited clinical effectiveness of the regimen [14]. Newer, direct acting antivirals (DAAs) for HCV, namely telaprevir and boceprevir, were only approved for the treatment of HCV in 2011 and sofosbuvir and simeprevir were most recently approved in late 2013 [15–18]. In addition, HIV has been in the forefront of public health issues for the past few decades and has been the focus of several government-sponsored initiatives. Examples of such initiatives include the U.S. President’s Emergency Plan for AIDS Relief (PEPFAR), funding through the Ryan White HIV/AIDS drug assistance program, and the CDC’s recommendations for HIV opt-out testing. The disparity between outcomes in HIV and HCV underscores the need to allocate more resources to HCV care in hopes that similar large-scale improvements can also be accomplished for this patient population.

This study is subject to some limitations. Foremost, this was a retrospective study using cross-sectional data. The observations only represent one specific point in time; a temporal relationship with long-term outcomes could not be determined. Medication information is not available in the NHDS; therefore, it was not possible to characterize treatment utilization (HCV antiviral therapy or HIV antiretroviral therapy). The data presented do not necessarily represent individual patients; rather the data represent individual hospitalizations. The study is unable to characterize or quantify the financial impact on the inpatient system regarding HIV, HCV, or HIV/HCV hospitalizations. Lastly, the study period (1996–2010) was selected to assess how inpatient care utilization and hospital outcomes changed during a time when both HAART and HCV combination antiviral therapy were available. However, this study does not capture recent outcomes reflective of newer HCV triple therapy regimens since the new DAAs were only approved for use in 2011 and 2013.

## Conclusions

Hospitalizations have declined more rapidly for patients with HIV infection (including HIV/HCV coinfection) than for patients with HCV monoinfection. This growing disparity between HIV and HCV underscores the need to allocate more resources to HCV care in hopes that similar large-scale improvements can also be accomplished for patients with HCV.

## Authors’ original submitted files for images

Below are the links to the authors’ original submitted files for images. Authors’ original file for figure 1 Authors’ original file for figure 2 Authors’ original file for figure 3
