# Supplementary figures and images for: National trends in hospitalization and mortality rates for patients with HIV, HCV, or HIV/HCV coinfection from 1996–2010 in the United States: a cross-sectional study
Source: BMC Infect Dis. 2014 Oct 10;14:536. doi: 10.1186/1471-2334-14-536 (PMC4287456; doi:10.1186/1471-2334-14-536)

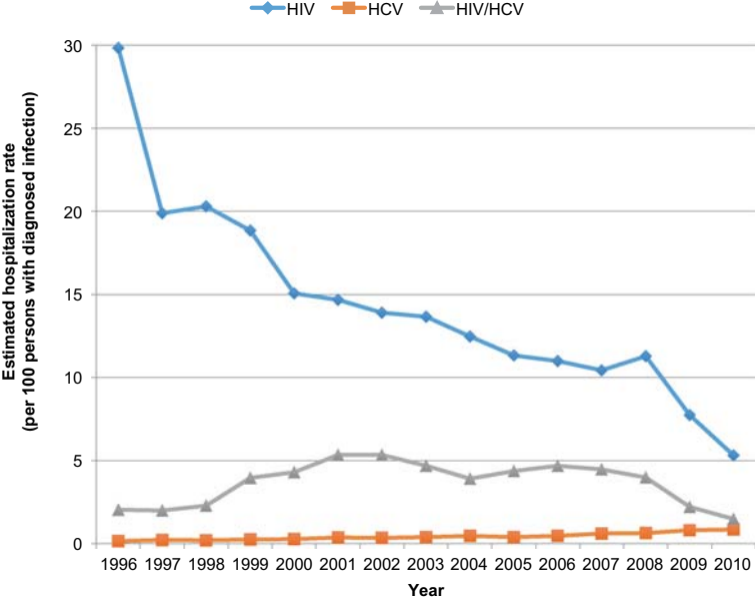

Supplement: Supplementary file 1 — Authors’ original file for figure 1 [file 12879_2014_3859_MOESM1_ESM.pdf]

**Age-Adjusted Hospital Mortality Rates (per 100,000 population)**

**Rate per 100,000 standard population**

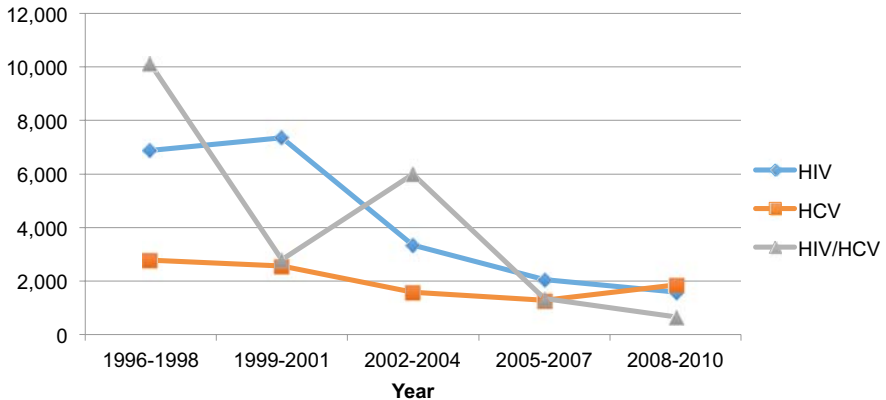

Supplement: Supplementary file 2 — Authors’ original file for figure 2 [file 12879_2014_3859_MOESM2_ESM.pdf]
