# Supplementary material for: National trends in hospitalization and mortality rates for patients with HIV, HCV, or HIV/HCV coinfection from 1996–2010 in the United States: a cross-sectional study
Source: BMC Infect Dis. 2014 Oct 10;14:536. doi: 10.1186/1471-2334-14-536 (PMC4287456; doi:10.1186/1471-2334-14-536)
Supplement: Supplementary file 3 — Authors’ original file for figure 3 [file 12879_2014_3859_MOESM3_ESM.pdf]

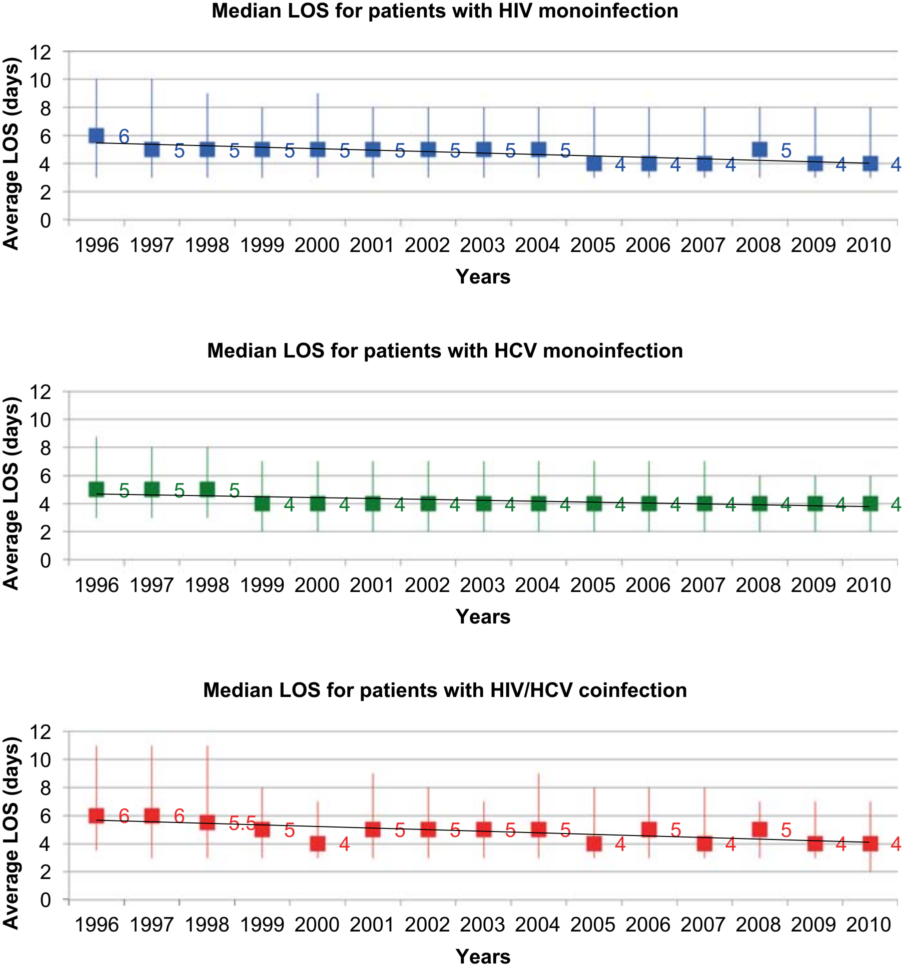

Median LOS for patients with HIV monoinfection
Average LOS (days)
0
2
4
6
8
10
12
1996
1997
1998
1999
2000
2001
2002
2003
2004
2005
2006
2007
2008
2009
2010
Years
6
5
5
5
5
5
5
5
5
4
4
4
5
4
4
Median LOS for patients with HCV monoinfection
Average LOS (days)
5
5
5
4
4
4
4
4
4
4
4
4
4
4
4
Years
Median LOS for patients with HIV/HCV coinfection
Average LOS (days)
6
6
5.5
5
4
5
5
5
5
4
5
4
5
4
4
Years
